# WEB-BASED PRESENCE FOR SOCIAL CONNECTEDNESS IN LONG-TERM CARE

**DOI:** 10.1093/geroni/igae098.1965

**Published:** 2024-12-31

**Authors:** Anna Garnett, Halyna Yurkiv, Richard Booth, Denise Connelly, Lorie Donelle

**Affiliations:** Western University, London, Ontario, Canada; Western University, London, Ontario, Canada; Western University, London, Ontario, Canada; Western University, London, Ontario, Canada; University of South Carolina, Columbia, South Carolina, United States

## Abstract

The COVID-19 pandemic and resultant restrictions on in-person gathering severely disrupted the social networks of older adults living in long-term-care homes (LTCH). Furthermore, the pandemic highlighted the need for embedded interventions such as web-based technology (WBT) to support social connectedness and well-being among older adults and their families. This qualitative study sought to explore how older adults living in LTCH, their family members and staff benefit from and envision WBT to support social connectedness. Semi-structured interviews were conducted with 22 family members, seven older adults and 10 staff across three LTCH. Data were analyzed using directed content analysis informed by Technology Acceptance Model. Participants predominantly used iPads, tablets, and phones to videoconference via Zoom, Teams, Facetime, or Skype. Older adults’ sense of social connectedness benefitted from their ability to visualize faces, observe non-verbal cues, perform lip reading, and follow the lives of their families outside of LTCH using WBT over conventional phone connections. In most cases, staff facilitated WBT use due to the older adults varied digital literacy, high rates of dementia and physical or visual limitations. Some family member participants found greater utility in videoconferencing technology called Portal and Alexa Echo Show which required limited navigation by the older adult. Henceforth, LTCH must adopt and implement technology that reflects the needs of people living in LTCH. In addition, adequate staffing, training, and funding is needed to enhance and sustain WBT use for the purposes of social connection among older adults in LTCH and their families.

